# Occupational Exposure and Health Impairments of Formaldehyde on Employees of a Wood Industry

**DOI:** 10.15171/hpp.2015.035

**Published:** 2016-01-30

**Authors:** Mohammad Javad Jafari, Abolfazl Rahimi, Leila Omidi, Mohammad Hassan Behzadi, Mohammad Hassan Rajabi

**Affiliations:** ^1^Occupational Health Engineering Department, Shahid Beheshti University of Medical Sciences, Tehran, Iran; ^2^Department of Environment and Energy, Science and Research Branch, Islamic Azad University, Tehran, Iran; ^3^Occupational Health Engineering Department, Tehran University of Medical Sciences, Tehran, Iran; ^4^Department of Statistics, Science and Research Branch, Islamic Azad University, Tehran, Iran; ^5^Doctorate of Laboratory Sciences, Golestan University of Medical Sciences, Gonbad, Iran

**Keywords:** Formaldehyde, Occupational Exposure, Symptoms, Blood Cell, Wood Industry

## Abstract

**Background:** Occupational exposure to formaldehyde may decrease white blood cell counts and change blood concentration. In this study, the influences of occupational exposure to formaldehyde on the number of white blood cells and blood concentrations were studied.

**Methods:** This case-control study was conducted in June of 2012 at North Wood Factory, Golestan Province, Iran. The US-NIOSH method No. 2541 was used to determine the occupational exposure of 30 workers of the production line (case group) and 30 administrative staffs (control group) to formalde­hyde. The number of white blood cells and blood concentration were determined using the normal blood count method and related indices. Demographic features as well as the symptoms of being exposed to formaldehyde were collected using a standard questionnaire.

**Results:** The occupational exposure of case group ranged from 0.50 ppm to 1.52 ppm. The prevalence of all studied symptoms from formaldehyde exposure in workers (2<median<5; range 1 to 5) was signifi­cantly different (P<0.001) towards the administrative staffs (median 1; range 1 to 4). The number of white blood cells in production line workers was not significantly different from those in administra­tive staff. The average blood concentration in the case group was significantly different from the con­trol group (mean difference= 0.9 [95% CI: 0.40-1.39];P=0.007).

**Conclusion:** Occupational exposure to formaldehyde changed the blood concentration of the studied workers but did not change the number of their white blood cells.

## Introduction

Exposure to toxic agents in various occupational environments is the most important issues in occupational health.^1^ Formaldehyde as a single carbon compound with chemical formula of O=C-H_2_ is a highly reactive and potent stimulus.^2^ Exposure to low levels (0.1ppm) of formaldehyde irritates eyes; nose and upper respiratory airways.^3,4^ Exposure to high concentrations may result in nerve palsy, impairment of pulmonary function and asthma.^5^ In prolonged exposures, cases of nasopharyngeal cancer^5^ as well as leukemia^6,7^ have been observed in humans. The known effect of formaldehyde in human cancer will significantly increase if in addition to nasopharyngeal cancer, the leukemia is also considered.^8^ The risk of lymphohematopoietic cancers especially myeloid blood cancer has been observed in industrial workers exposed to formaldehyde.^9^ A possible relationship between the lymphohematopoietic cancers and the level of exposure to the formaldehyde has been reported.^10^ The prevalence of myeloid leukemia is higher than other leukemia.^10,11^

The question remains whether the formaldehyde with such a reactive specification reaches to the bone marrow and the primitive hematopoietic cells which are the targets in case of cancer or not.^12,13^ One of the clinical consequences of damaging primitive hematopoietic cells is the reduction of circulated red and white blood cells (WBC) as well as the number of plackets.^14^ Formaldehyde can mutate primitive cells leading to gene mutation or broken chromosomes which may result in cancer.^15-17^ Inhalation of formaldehyde may damage the liver of some animal species.^18^

In 1995, U.S. Environmental Protection Agency (US-EPA)^19^, and in 2004, the International Agency for Research on Cancer (IARC) recommended formaldehyde as a group A ‏" human carcinogen material‏". ^20,21^ In 2011, in its 12^th^ report, IARC announced that prolonged exposure to formaldehyde can lead to nasopharyngeal cancer and a type of leukemia.^22,23^ American Conference of Governmental Industrial Hygienists (ACGIH) has not announced the 8**-**h occupational exposure limit for formaldehyde but has recommended the ceiling level of 0.3ppm for it.^24^

Previous studies have reported the exposure of workers in particle board production line to be from 0.1 ppm to 1 ppm^25,26^ that is higher than the determined levels for the prevention of leukemia. Thus, in processes such as particleboard and plywood production, the workers are likely to experience changes in the number of white blood cells and the concentrations of their blood. In this study, we examined the association between WBC count and blood serum concentration of workers with their occupational exposure to formaldehyde in a wood factory.

## Materials and Methods

### Study participants

This case-control study was conducted in June of 2012 at North Wood Factory, Golestan Province, northern Iran. Cochran's sample size formula was used to calculate the sample size.

The α was set at 0.05 and the level of precision was 0.1. Thirteen workers of the production line (case group) and 30 administrative staffs (control group) were participated in the study according to the set of criteria. Non-smoking, full time workers with at least one**-**year experience in the studied factory with no-infectious diseases affecting the WBC count and blood concentration were included in the study. The participants with no full corporation and those unwilling to continue the study were excluded. Maintenance personnel and those who were not permanently exposed with formaldehyde were also excluded. Eight samples were excluded from the study for confounding variables. They included three cases due to smoking, one case due to lung disease, one case due to renal disease and 3 cases due to severe stress of the participant during the test.

To eliminate possible confounding parameters, the administrative staffs of the same company who were largely in terms of lifestyle and diet type similar to the case group were determined as the control group.

Demographic variables including age, work experience, smoking habits, health condition regarding the WBC count, blood serum and symptoms related to formaldehyde exposures, were collected by a standard questionnaire. The objectives of the study were explained to the participants prior to their start completing the questionnaire.

### Sampling of formaldehyde

US-NIOSH method No. 2541 was applied for air sampling. For this purpose, the subjective SKC**-**UK sampling pumps connected to a special sorbent tube (ST) through a polyethylene tube was used. All sampling pumps were calibrated in rate of 0.1 l/min by a Rota**-**meter, prior to start air sampling. Sampling was conducted within volumes of 1 to 36 liters in various stations according to the recommended method. Air sampling was performed from 8:30 to 14:30 in the morning shift, where maximum volumes of working were expected.^27^ A total number of 60 air samples was taken from the worker's breathing zone. The sorbent tubes were taken off their circuit and capped after sampling. All samples were left in cover and special holder (ice jar) keeping them away from any moisture, shock and light while transferring to the laboratory. Gas chromatography (GC 6890) using flame ionization detector (FID) was used to analyze the samples.^27^

### Blood sampling

Blood samples were taken one hour before the end of working shift in the health care center of the factory by a nurse (monitored by a medical doctor) from workers arm vein. Blood samples were refrigerated until tested. To measure the peripheral blood parameters, the cell counter set of Sysmex model made in Japan was used. For typical investigation of peripheral blood parameters, the common method of blood cell count and the related indices were used. The required blood for cell counts was 2 ml. The blood was taken on ethylenedi**-** amine**-**tetra acetic acid (EDTA) in order to avoid its coagulation.^28^ Moreover, to check white blood cell morphology, a blood smear was prepared on slides from each sample, stained with Giemsa color, and tested using Zeiss microscope.

### Ethical Issues

Ethical aspects were concerned due to the aggressive nature of some tests. For this purpose, after explanation of the tests including the purpose of the study plan, a written consent was received from each participant prior to blood sampling. The provisions of the Declaration of Helsinki for medical research involving human subjects were considered. The proposal of the study was approved by the Ethical Committee of Research and Science Branch of Islamic Azad University. Meanwhile, the results of the tests were kept completely confidential.

### Statistical analysis

Statistical analyses were performed using the SPSS 16 for Windows (SPSS Inc., Chicago, IL, USA). Statistical *t*-test was used to compare the mean age and working experience of workers in the case and control groups. The Mann–Whitney U**-**test was applied to compare the education levels of workers in the case and control groups. The prevalence of irritating symptoms in both case and control groups were compared with Friedman test. Multiple regression analysis was performed to assess statistical distribution of factors influencing the white blood cell counts and blood concentration in studied groups. In all analyses *P*<0.05 was considered as significant.

## Results

### Demographic characteristics

The age of the case group ranged from 27 to 55 yr with an average and standard deviation of 37.23 (SD 9.07) yr. The control group was in the range of 25 to 58 yr old with an average and standard deviation of 41.53 (SD 7.86) yr. The highest number of participants in the case group (11 workers) was in the 20**-**30 age group. Among subjects in the control group, 14 of them were in the 40**-**50 age group. Statistical t**-**test showed no significant difference between the average age of case and control groups (*P*=0.055).

Working experience of the case group ranged from 2 to 22 years, with an average and standard deviation of 10.57 (SD 6.91) yr. The working experience of the control group was in the range of 3 to 26 yr with an average and standard deviation of 15.27 (SD 7.24) yr. The highest number of participants in the case group (12 workers) had work experience of < 5 years. Among subjects in the control group, 9 of them had work experience of 15**-**20 years. The statistical *t-*test showed a significant difference between the average working experience in case and control groups (*P*=0.013).

Education levels of case and control groups are compared with Mann–Whitney U**-**test. The highest number of participants in the case group (16 workers) had diploma. Among subjects in the control group, 11 of them had Bachelor of Science (BS) degree. The results of statistical test showed that education level of groups are significantly different (*P* = 0.001).

### Symptoms of exposure to formaldehyde

**Fig 1:**
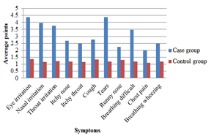
Distribution of exposure symptoms to formaldehyde

The Mann–Whitney U**-**test showed that the average points obtained for prevalence of symptoms from formaldehyde exposure in workers (2<median<5; range 1 to 5) was significantly different (*P*<0.001) towards the administrative staffs (median 1; range 1 to 4). Average points obtained for prevalence of symptoms in the case group were higher than those of the control group (Figure 1). The Friedman statistical test also showed that the prevalence of irritating symptoms in both case and control groups was significantly different (*P*<0.001).

Figure 1 shows the significant differences in the prevalence of irritating symptoms in the case and control groups. The prevalence of irritating symptoms is compared in Table 1.

**Table1:** Prevalence of irritating symptoms of formaldehyde exposure

**Symptom**	**Median (min– Max)**	
	**Case group**	**Control group**
Eye irritation	5 (3**-**5)	1 (1**-**4)
Nasal irritation	4 (1**-**5)	1 (1**-**3)
Throat irritation	4 (1**-**5)	1 (1**-**3)
Itchy nose	2 (1**-**5)	1 (1**-**2)
Itchy throat	2 (1**-**5)	1 (1**-**4)
Cough	3 (1**-**5)	1 (1**-**3)
Tears	5 (2**-**5)	1 (1**-**3)
Runny nose	2 (1**-**5)	1 (1**-**4)
Breathing difficult	3 (2**-**5)	1 (1**-**3)
Chest pain	2 (1**-**5)	1 (1**-**2)
Breathing wheezing	2 (1**-**5)	1 (1**-**3)

### Exposure to formaldehyde

The occupational exposure of control group to formaldehyde was 0.00 ppm, while the exposure of case group ranged from 0.50 ppm to 1.52 ppm with a median of 1.30 ppm. According to the results of Mann–Whitney U**-**test, the average occupational exposure to formaldehyde in control and case groups was significantly different (*P*=0.001).

Exposure level of all control samples (administrative staff) was lower than the recommended carcinogenesis of 0.1ppm. All workers in production line are experiencing higher exposure levels than the ceiling level of 0.3ppm recommended by US**-**ACGIH. About 57% of the workers had an exposure level of 1.25**-**1.50ppm. About 17% and 13% of them had exposure levels of 1.00**-**1.25 ppm and 0.50**-**0.75 ppm, respectively.

### The number of white blood cells

The number of WBC in control group ranged from 4.90×10^3^/µl to 10.80×10^3^/µl with an average ± standard deviation number of 7.07 ± 1.29×10^3^/µl. The white blood cell counts in case group ranged from 4.70×10^3^/µl to 10.70×10^3^/µl with an average ±standard deviation number of 6.78 ± 1.49×10^3^/µl. The result of statistical test showed no significant difference between the mean white blood cell count in the case and control groups. Pearson correlation coefficient calculated for case group also showed no meaningful relation between the exposure levels and the number of white blood cells in case group (r=0.128*; P*=0.498).

Table 2 shows the possible factors affecting the white blood cell counts in case and control groups. These factors include age, work experience and education level, evaluated using multiple regression test. There was no significant relationship between these factors and the average number of WBC in both case and control groups.

### Blood concentration

The minimum, maximum, mean ± standard deviation of blood concentration in the control group was 12.30g/100cm^3^, 15.60g/100cm^3^, and 14.25±0.99g/100cm^3^, respectively. Minimum, maximum, average ± standard deviation of the blood concentration in case group was 12.70g/100cm^3^, 16.00g/100cm^3^, and 15.00 ± 0.91g/100cm^3^, respectively. *T-test* showed that the average concentration of the blood in case group was significantly different from the mean blood concentration of the control group (mean difference= 0.9 [95% confidence interval (CI): 0.40-1.39]; *P*=0.007).

**Table 2:** Statistical distribution of factors influencing the white blood cell counts in studied groups

**Factor**	**Control group**			**Case group**		
	**Beta**	***t***	***P***	**Beta**	***t***	***P***
Age (yrs.)	**-**0.177	**-**0.509	0.615	0.036	0.094	0.926
Experience (yrs.)	**-**0.035	**-**0.102	0.920	0.330	0.852	0.402
Education (level)	**-**0.078	**-**0.414	0.682	**-**0.178	-0.957	0.347

Pearson correlation coefficient calculated for case group also showed that there was a meaningful relation between the exposure levels and blood concentration in case group (r=**-**0.473 *P*=0.015). Statistical distribution of factors influencing the blood concentration is shown in Table 3. They include age, work experience and education level. Statistical test using multiple regressions showed no significant difference between these factors and blood concentrations in studied groups.

**Table 3:** Statistical distribution of factors influencing the blood concentration in studied groups

**Factor**	**Control group**			**Case group**		
	**Beta**	***t***	***P***	**Beta**	***t***	***P***
Age (yrs.)	**-**0.493	**-**1.473	0.152	**-**0.004	**-**0.011	0.991
Experience (yrs.)	0.606	1.808	0.082	**-**0.204	**-**0.532	0.599
Education (level)	0.063	0.337	0.739	**-**0.133	**-**0.710	0.484

## Discussion

Participants in this study were all working in the same factory and in terms of social, and demographic characteristics such as gender, age, lifestyle and diet were almost similar. However, the work experience and the education level of case and control groups were significantly different. The education level of control group was significantly higher than the workers of production line.

The workers of production line (case group) were experiencing significantly higher levels of formaldehyde than the administrative staffs (control group). Formaldehyde exposure level of all participants in the case group was higher than the ceiling level of 0.3ppm recommended by US ACGIH^24^, while 90% of them were imposed with exposure levels of higher than 0.5ppm. The number of WBC decreases as the age goes up.^29^ Since the work experience represents the age in some way thus, the number of white blood cells are expected to decrease as the work experience increases as well. In industrial plants, usually higher educated personal have supervising and administrative duties expecting to have less exposure to the air pollutants. Thus, the age, work experience, and the education level may be considered as the influencing factors on blood cell counts of the workers exposed to formaldehyde.

Previous studies have reported the exposure level of workers in particleboard production line to be from 0.1ppm to 1ppm^25,26^ that are lower than the exposure levels of workers measured in this study (e.g. 0.50 ppm to 1.52 ppm). The measured levels of exposure to formaldehyde for case group were well higher than the determined levels (0.1ppm) for the prevention of leukemia.^28,30^

However, the high solubility of formaldehyde in water as well as its respiratory, eye and mucus tubes irritation is causing symptoms such as runny eyes, runny nose, coughing and wheezing while breathing it. In this study, these stimulatory signals were also examined. The prevalence of all irritation symptoms in case group (e.g. workers of production line) was significantly higher than the prevalence of irritation symptoms in the control group.

Tearing had the highest and the chest pain had the lowest prevalence in production line workers. These results are in consistence with the results described earlier, which showed a high prevalence of eye irritation symptoms among exposed workers.^31^

The number of white blood cells in adults ranged from 4.5×10^3^/µl to 10×10^3^/µl. The number of white blood cells in case group compared to this range, shows that it is shifted down a little but it is not significantly being reduced compared to the control group.

The number of WBC could be considered as an indicator of formaldehyde exposure level^32^ that is difficult to confirm it in low exposure levels similar to those experienced in present study. No significant relationship was observed between the number of WBC of case and control group.

The results of this study indicated that long**-**term exposures to low levels of formaldehyde do not change the number of WBC. The finding is in agreement with previous findings.^33^ The exposure to the formaldehyde significantly (*P*=0.0016) reduced the number of WBC of Chinese workers^34^ which contradicts with the results of the present study. In order to eliminate possible confounding factors, 8 cases of confounding variables were excluded from the analysis.

This study shows that formaldehyde exposure significantly increased the blood concentration of workers, which is consistent with previous study in China.^4^ The increased occupational exposure to formaldehyde caused the blood viscosity reduction.^4^ Inhaled formaldehyde causes hypoxia which decreases tissue oxygen. In reaction to this condition, hypoxia is secreted by the kidneys through erythropoietin hormone frequently. This stimulates the production of red blood cells in the bones and consequently increases the amount of hemoglobin. As the blood viscosity increase, this difference is created.^35,36^ but with higher exposure, the reactive power of the body is expected to decrease leading to lower blood concentration. Formaldehyde effects only at the beginning of its contact.^36^

A limitation of this study is that the numbers of cases and controls were relatively small. However, these findings are limited by the use of a cross sectional design. This research has thrown up many questions in need of further investigation. Further work needs to be done to establish whether occupational exposure to formaldehyde affects human bone marrow cells. More broadly, research is also needed to determine the effect of formaldehyde exposure on the differential white blood cell counts.

## Conclusion

According to the results of the study, the number of white blood cells is not an appropriate indicator of long term exposure to formaldehyde. However, it is necessary to select reliable indicator as a biological indicator of exposure to formaldehyde with additional testing. One of the more significant findings to emerge from this study is that low**-**level formaldehyde exposure caused an increase in the concentration of the blood.

## Acknowledgement

This article is extracted from A Rahimi's thesis supervised by Dr MJ Jafari. The project was financially supported by the North Wood Industry Co. The authors wish to appreciate greatly their financial supports. We also, thank the Shohada Hospital of Gonbad for their technical supports.

## Conflict of interest

The authors announce no conflict of interest.
